# High Yield Production of a Soluble Human Interleukin-3 Variant from *E. coli* with Wild-Type Bioactivity and Improved Radiolabeling Properties

**DOI:** 10.1371/journal.pone.0074376

**Published:** 2013-08-26

**Authors:** Timothy R. Hercus, Emma F. Barry, Mara Dottore, Barbara J. McClure, Andrew I. Webb, Angel F. Lopez, Ian G. Young, James M. Murphy

**Affiliations:** 1 Centre for Cancer Biology, SA Pathology, Adelaide, South Australia, Australia; 2 The Walter and Eliza Hall Institute of Medical Research, Parkville, Victoria, Australia; 3 Department of Medical Biology, University of Melbourne, Parkville, Victoria, Australia; 4 Department of Molecular Bioscience, John Curtin School of Medical Research, The Australian National University, Canberra, Australian Capital Territory, Australia; Centro Nacional de Biotecnologia - CSIC, Spain

## Abstract

Human interleukin-3 (hIL-3) is a polypeptide growth factor that regulates the proliferation, differentiation, survival and function of hematopoietic progenitors and many mature blood cell lineages. Although recombinant hIL-3 is a widely used laboratory reagent in hematology, standard methods for its preparation, including those employed by commercial suppliers, remain arduous owing to a reliance on refolding insoluble protein expressed in *E. coli*. In addition, wild-type hIL-3 is a poor substrate for radio-iodination, which has been a long-standing hindrance to its use in receptor binding assays. To overcome these problems, we developed a method for expression of hIL-3 in *E. coli* as a soluble protein, with typical yields of >3mg of purified hIL-3 per litre of shaking microbial culture. Additionally, we introduced a non-native tyrosine residue into our hIL-3 analog, which allowed radio-iodination to high specific activities for receptor binding studies whilst not compromising bioactivity. The method presented herein provides a cost-effective and convenient route to milligram quantities of a hIL-3 analog with wild-type bioactivity that, unlike wild-type hIL‑3, can be efficiently radio-iodinated for receptor binding studies.

## Introduction

Human interleukin-3 (hIL-3) is a four-helix bundle, short chain cytokine that is widely expressed *in vivo*, principally by hematopoietic cells, such as activated T-lymphocytes, mast cells and basophils [1]. This cytokine serves an important role in the regulation of proliferation, differentiation, survival and activation of a range of hematopoietic cell types, including progenitor cells, dendritic cells, basophils and mast cells [2–4]. In particular, IL‑3 is known to stimulate the production and function of basophils and mast cells in the context of allergic inflammation *in vivo* [5,6], and is commonly used as a stimulus to culture these cell types *in vitro* (for example, 7–9). Owing to its importance in immune cell stimulation and implication in the pathogenesis of acute myeloid leukemia and chronic myeloid leukemia [10–13], IL-3 signaling has emerged as a potential therapeutic target for the treatment of allergic diseases, including asthma, and hematopoietic malignancies.

The effects of IL-3 on target cells are initiated by IL-3 binding to a transmembrane receptor system composed of an IL-3-specific α-subunit (IL-3Rα) and a common β-subunit (βc) shared with the related cytokines, IL-5 and GM-CSF [14–16]. Engagement of these cell surface receptors by IL-3 leads to transmission of a signal across the cell membrane, *via* a poorly understood process, activation of intracellular signaling networks and subsequent cellular responses. A bottleneck in our efforts to characterize hIL-3 engagement of its receptor *in vitro* has been a paucity of efficient methods for hIL-3 production from *E. coli*. Wild-type recombinant hIL-3 expressed in *E. coli* exhibits limited solubility [17] and has been typically produced by oxidative refolding of material expressed in inclusion bodies, even by commercial suppliers. Here, we describe an economical, rapid and simple method to express and purify milligram quantities of soluble hIL-3 from *E. coli*. In addition to affording high yields (typically >3mg/L of shaking microbial culture), the resultant recombinant hIL-3 bears a non-native tyrosine at the N-terminus to allow efficient ^125^I-radiolabelling for receptor binding studies. Despite truncation of the native sequence to enhance solubility, as described in the pioneering work of Olins et al. [17], and the introduction of the non-native N-terminal tyrosine, the hIL-3 expressed and purified from the soluble fraction of *E. coli* lysates using our method and commercially-sourced recombinant hIL-3 preparations were equipotent growth stimuli in TF-1 cell proliferation studies. Therefore, the method described herein provides a robust and convenient strategy to produce milligram quantities of fully bioactive soluble hIL‑3 for a broad range of laboratory applications, including hematopoietic cell culture and molecular characterization of hIL-3 receptor binding.

## Materials and Methods

### Expression construct

A cDNA encoding hIL-3 residues 13-125, including the W13Y mutation, was amplified from an IMAGE clone template (IMAGE: 6971773; NCBI accession: BC066272) with *Pfu* polymerase using the primers: 5’ CGC GGa TCc tAT gtt aac tgc tct aac atg atc gat g; 3’ ATAAGAATGCGGCCG cTA ctg ttg agc ctg cgc att ctc. The PCR product was digested with BamHI and NotI restriction endonucleases before ligation into the corresponding restriction sites in the vector, pETNusH HTb [18], a derivative of pETM30 bearing a kanamycin-resistance marker. The insert was verified by Big Dye Terminator sequencing.

### Protein expression and purification from *E. coli*

*E. coli* BL21 CodonPlus (DE3)-RIPL cells transformed with the expression construct described above were cultured at 37°C in 2 L flasks containing 0.6 L of TYH medium (20 g tryptone, 10 g yeast extract, 5 g NaCl and 1 g MgSO_4_ per litre with 46 mM HEPES pH 7.4) and supplemented with 50 µg/ml kanamycin. Once the optical density at 600 nm reached 0.6-0.8, cultures were adjusted to 1.5 mM isopropyl β-D-1-thiogalactopyranoside (IPTG) and incubated at ~23°C with shaking for ~16 hours. We found that induction for ~16 hours at 18-25°C gave comparable yields, although higher expression temperatures were not tested. Cells were harvested by centrifugation and lysed by sonication in NL buffer (0.2 M NaCl, 50 mM Na phosphate pH 8.0, 10% v/v glycerol, 0.05% v/v Na azide) freshly supplemented with 20 mM imidazole, 0.05% v/v Tween 20 and 1 mM phenylmethylsulfonyl fluoride (PMSF). The lysate was clarified by centrifugation at 26,800x*g* for 30 minutes followed by 0.45 µm filtration of the supernatant. Crude lysate was bound at 4°C and 2 ml/min to a 10 ml column of Ni-NTA Agarose (Qiagen) equilibrated in NL buffer that had been packed in an XK-26 column holder (GE Healthcare). The Ni-NTA agarose was washed in 200 ml NL buffer containing 20 mM imidazole at 5 ml/min and the bound NusA-His_6_-hIL-3 (13-125; W13Y) fusion protein eluted in 100 ml NL buffer containing 250 mM imidazole at 5 ml/min and collected in 10 ml fractions. Fractions containing purified NusA-His_6_-hIL-3 (13-125; W13Y) fusion protein were pooled and concentrated to a final volume of < 20 ml using Vivaspin 20, 30,000 molecular weight cut-off devices (Sartorius). The concentrated fusion protein was dialysed against 50mM Tris-HCl pH 7.4, adjusted to 1 mM dithiothreitol (DTT), 0.5 mM EDTA and mixed with purified TEV protease, produced in-house, at 23°C for ~16 hours. The amount of TEV protease used was empirically determined for each batch of the NusA-His_6_-hIL-3 (13-125; W13Y) fusion protein to achieve complete cleavage. Typically, complete cleavage required up to 0.1 mg TEV protease per 10 mg NusA-His_6_-hIL-3 (13-125; W13Y).

The TEV protease-digested NusA-His_6_-hIL-3 (13-125; W13Y) fusion protein was fractionated by size exclusion chromatography (SEC) using a Superdex 200 column (26 mm x 600 mm, GE Healthcare) fitted with a 10 ml Super Loop and operated at 4°C with a flow rate of 2 ml/min. SEC was performed using a running buffer composed of 150 mM NaCl, 50 mM Na phosphate pH 7.0. SEC fractions containing hIL-3 were established by SDS-PAGE analysis with Coomassie Blue staining and then pooled, with a portion sterilized using Spin-X filters (Corning).

In some cases, SEC purified hIL-3 (13-125; W13Y) was further purified by reversed-phase chromatography. Glacial acetic acid was added to the SEC purified hIL-3 (13-125; W13Y) to a final concentration of 1% (v/v) while trifluoroacetic acid was added to a final concentration of 0.1% (v/v). The sample was applied to an Aquapore RP300 reversed-phase column (4.6 mm x 100 mm) in buffer A (0.1% v/v trifluoroacetic acid in water) via a 2 ml injection loop fitted to a Breeze 2 semi-preparative HPLC system (Waters). Bound proteins were eluted over 60 minutes using a linear gradient from 100% buffer A to 100% buffer B (0.085% v/v trifluoroacetic acid in acetonitrile) at 1 ml/min with the collection of 1 ml fractions into tubes containing 20 µl 2M Tris. Fractions containing purified hIL-3 (13-125; W13Y) were pooled, lyophilized, resuspended in PBS and sterilized using Spin-X filters.

Purified hIL-3 (13-125; W13Y) was quantitated by analytical SEC using a Superdex 200PC column (3.2 mm x 300 mm, GE Healthcare) and a 50 µl loop operated at 25°C and 40 µl/min with a running buffer composed of 150 mM NaCl, 50 mM Na phosphate pH 7.0. The area under the hIL-3 peak was integrated by using the calculated extinction coefficient of 0.644 M^-1^ cm^-1^. The concentrations of commercially-sourced hIL-3 used to compare the bioactivity of hIL-3 (13-125; W13Y) was specified by the manufacturers.

### Mass spectrometry analysis

hIL-3 (13-125; W13Y) was digested with trypsin using the FASP protocol [19] and eluted peptides were injected and separated by nano flow reversed-phase liquid chromatography on a nano LC system (1200 series, Agilent) using a nanoAcquity C18 150 mm × 0.15 mm I.D. column (Waters) with a linear 45 minute gradient from 5 to 100% buffer B set at a flow rate of 1.2 µl/min (Buffer A: 0.1% Formic acid in Milli-Q water; Buffer B: 0.1% Formic acid, 80% acetonitrile (Mallinckrodt, Baker) 20% Milli-Q water). The nano HPLC was coupled on-line to a Q-Exactive mass spectrometer equipped with a nano-electrospray ion source (Thermo, Fisher Scientific) for automated MS/MS. The Q-Exactive was run in a data-dependent acquisition mode with the full scan resolution set at 30,000 and the top-ten multiply charged species selected for fragmentation using the high energy collision disassociation (HCD) (single charged species were ignored). Fragment ions were analysed with the resolution set at 17,500 and the ion threshold set to 1e5 intensity. The activation time was set to 30 ms and the normalized collision energy set to 24. Raw files consisting of full-scan MS and high resolution MS/MS spectra were converted to the MGF data format with Proteome Discoverer 1.4 and searched against UniProt database (2013/02) including the hIL-3 analog sequence, limiting the search to human taxonomy using Mascot v 2.4. Mascot parameters for each search included Trypsin/no P enzyme with three missed cleavages, a fixed modification in the form of carbamidomethyl at Cys residues and variable modifications of Acetyl at protein N-Terminal and oxidation at Met residues. Spectra were searched with a mass tolerance of 20 ppm in MS mode and 40 mmu in MS/MS mode.

### IL-3 Bioactivity Assays

The proliferative activity of hIL-3 (13-125; W13Y) was assayed using the GM-CSF, IL-3-dependent human erythroleukaemia cell line, TF-1 [20] according to established protocols (for example, 21). Cells (5 x 10^4^ per well) were incubated with serially-diluted hIL-3 in 96-well plates for 40 hours before being pulsed with 0.25 µCi per well of [6-^3^H] thymidine (PerkinElmer) for 6 hours. Cells were harvested onto glass fibre filters and washed extensively before scintillation counting in liquid scintillation fluid on a Top Count NXT (PerkinElmer).

### IL-3 Binding Assays

Purified hIL-3 (13-125; W13Y) was radio-iodinated using Pierce Pre-Coated Iodination tubes (Thermo Scientific) according to established protocols [22,23]. COS cells were transfected by electroporation with expression plasmids encoding the human IL-3Rα subunit (pSG5: IL‑3Rα) and the human βc subunit (pSG5: βc). Binding assays using COS cells transiently expressing IL-3 receptors was performed as previously described [24] and the data analysed using EBDA-LIGAND software (KELL; Biosoft).

## Results

### Expression construct design

**Figure 1:**
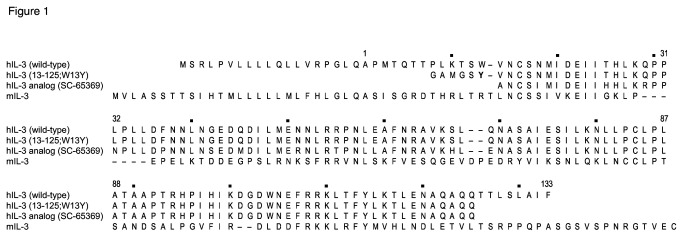
Alignment of amino acid sequences of human and mouse IL-3. The amino acid sequences of full length wild-type hIL-3, hIL-3(13-125; W13Y), the hIL-3 analog SC-65369 [41], and wild-type full length mouse IL-3 were manually aligned owing to low homology between mouse and human IL-3 (29% identity). Numbers above the sequence refer to the mature form of full length hIL-3 with dots above every 10^th^ residue. The sequences shown in gray for full length hIL-3 and full length mouse IL-3 are signal peptides that are cleaved during secretion. The key substitution, W13Y in hIL-3(13-125; W13Y), is shown in bold text and highlighted.

In an attempt to produce hIL-3 in the soluble fraction of *E. coli* lysates rather than in inclusion bodies, we prepared a fusion protein with NusA-His_6_ [18], (Figure 1). To this end, we PCR amplified a fragment encoding the four-helix bundle core of hIL-3, residues 13-125, and ligated into the vector, pETNusH, as an in-frame fusion C-terminal to the 55kDa solubility tag, NusA, and a His_6_ affinity tag to allow fusion protein purification by Ni^2+^ chromatography. Between the His_6_ tag and the hIL-3 sequence is a vector-encoded TEV protease recognition site. TEV protease cleavage of the NusA-His_6_ tag from hIL-3 introduces a non-native GAMGS at the N-terminus owing to a cloning artifact. We chose to incorporate a TEV protease cleavage site, since this enzyme can be readily prepared in the laboratory to high yields [25] and cuts with high efficiency and sequence specificity unlike other enzymes, such as thrombin and Factor Xa. Importantly, TEV protease offers the flexibility of cleaving substrates at 25°C, typically within 2 hours, or at 4°C overnight should the target protein be temperature sensitive.

Full length hIL-3 is expressed from mammalian cells as a pro-protein that is cleaved during secretion (signal peptide shown in grey in Figure 1) to yield a mature polypeptide, the first residue of which we have numbered as residue 1 (Figure 1). Prior studies have established that truncation of hIL-3 at either terminus enhances its solubility without compromising bioactivity [17] and the enhanced solubility of a truncated and highly-mutated hIL-3 analog, SC-65369 (Figure 1), allowed its solution structure to be determined [26]. Although the sequence of our hIL-3 protein is wild-type, but truncated at both N- and C-termini, we introduced a tyrosine in place of Trp13 (highlighted in Figure 1) in our expression construct to facilitate radio-iodination for receptor binding assays.

### Purification of hIL-3(13-125; W13Y)

**Figure 2:**
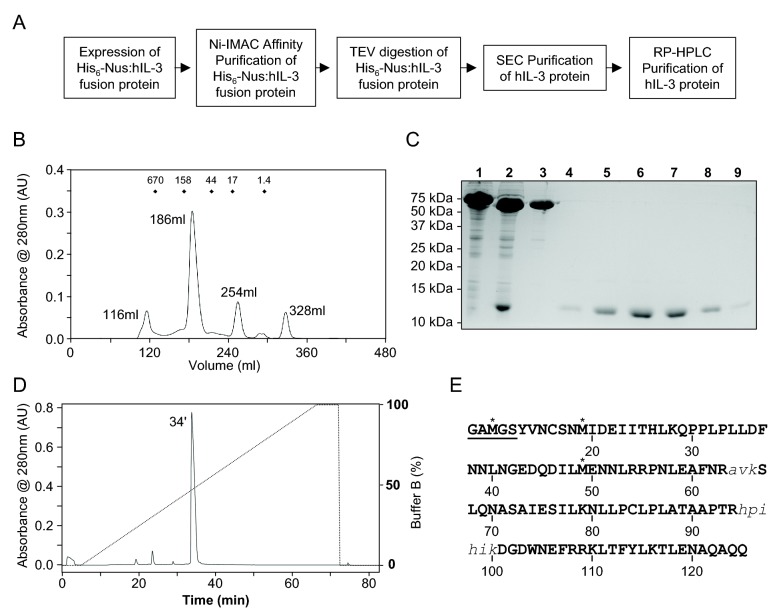
Purification of the hIL-3(13-125; W13Y) analog. A) Workflow diagram illustrating the purification protocol for hIL-3(13-125; W13Y). B) Elution profile of the TEV protease digested, NusA and hIL-3 analog mixture, following size exclusion chromatography (SEC) using a Superdex 200 column (26 mm x 600 mm) operated at 2 ml/min at 4°C with 50 mM sodium phosphate pH 7.0, 150 mM NaCl as running buffer. Free NusA eluted at ~186 mL and the hIL-3 analog eluted at 254 mL. The first peak at 116 mL contains aggregates while we suspect the last peak at 328 mL contains DTT from the digest. Molecular weight (kDa) marker elution positions are marked as dots above the elution profile. C) Analysis of hIL-3 analog purification by 15% acrylamide reducing SDS-PAGE with Coomassie Blue staining. NusA: hIL-3(13-125; W13Y) fusion protein was isolated by Ni-chromatography (Lane 1) prior to cleavage by TEV protease (Lane 2) to yield free NusA (55kDa) and the hIL-3 analog (13.4kDa). Fractions containing the hIL-3 analog that eluted around 254ml during SEC are shown (Lanes 4-9), illustrating that the hIL-3 analog was purified free from NusA (Lane 3). D) The SEC purified hIL-3 analog was applied to an Aquapore RP300 reversed-phase column (4.6 mm x100 mm) and bound proteins eluted using a 0-100% gradient of acetonitrile in 0.1% trifluoroacetic acid. The hIL-3 analog eluted at 34 min (~50% acetonitrile). E) Purified hIL-3(13-125; W13Y) was subjected to tryptic digestion and tandem mass spectrometry. The sequences of peptides not identified in this analysis are shown as lowercase italics. Asterisked methionine residues were oxidized. Sequence arising from the NusA-His_6_ fusion overhang after TEV protease cleavage is underlined, while the residues are numbered according to the mature, full-length IL-3 reference sequence.

We developed a method to prepare hIL-3 to high-purity and yield (Figure 2A). The expression of NusA-His_6_ fused hIL-3(13-125; W13Y) was carried out in *E. coli* BL21 Codon Plus (DE3) RIPL, followed by cell lysis and Ni^2+^-chromatography. Following cleavage of the fusion protein with TEV protease, the mixture was concentrated and the hIL‑3(13-125; W13Y) separated from uncut fusion protein, the NusA-His_6_ tag and TEV protease using preparative SEC. The molecular mass difference between NusA and hIL-3 was sufficiently large (55kD vs. 13.4kD) to permit resolution by Superdex-200 26/60 SEC, with NusA elution at 186 mL and hIL‑3 at 254 mL (Figure 2B). While the hIL-3 fractions were of high purity as gauged by SDS-PAGE with Coomassie Blue staining with no detectable NusA or TEV protease present (Figure 2C), we proceeded to purify hIL-3 to homogeneity using reversed-phase high-performance liquid chromatography (RP-HPLC; Figure 2D). RP-HPLC was performed with a gradient of 0 to 100% acetonitrile with hIL-3 elution occurring at ~50% acetonitrile. As evidenced by the RP-HPLC chromatogram shown in Figure 2D, negligible quantities of proteinaceous contaminants were present in hIL-3 following SEC, underscoring that RP-HPLC can be considered a polishing step but does not vastly enhance the purity of hIL-3. Routinely, the yields per litre of shaking bacterial culture exceed 3mg, although in some instances we have obtained up to 7mg of purified hIL-3(13-125; W13Y) per litre of culture.

We verified the correct sequence of purified hIL-3(13-125; W13Y) protein and confirmed the introduced modifications by performing mass spectrometry. Purified protein was subjected to tryptic digestion and tandem mass spectrometry analysis. As summarized in Figure 2E, >90% sequence coverage was observed, verifying the composition of the purified product. The identified peptides are shown in Table S1.

### Characterization of bioactivity relative to commercially-sourced hIL-3

**Figure 3:**
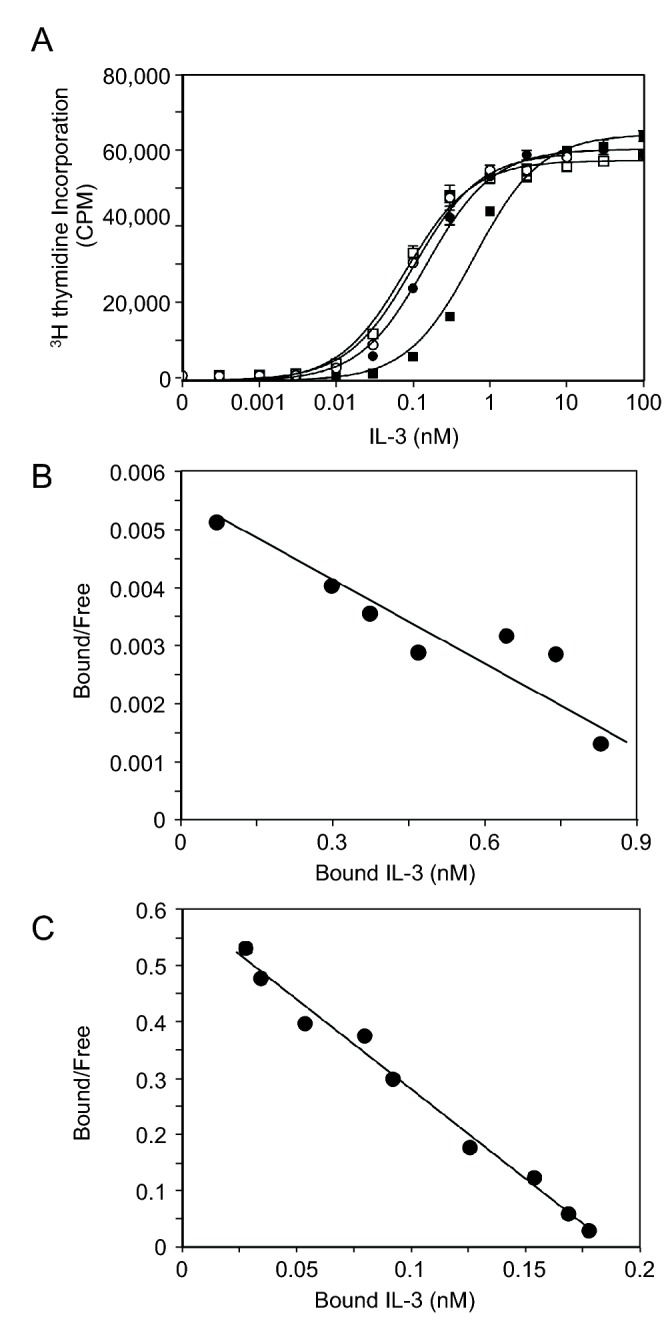
Functional activity of the hIL-3(13-125; W13Y) analog. A) TF-1 cell proliferation was assessed using titrations of hIL-3(13-125; W13Y) purified by SEC only (open squares) or purified by SEC plus RP-HPLC (open circles). As controls, commercial recombinant hIL-3 from supplier A (black circles) and B (black squares) were also included. Each value represents the mean of triplicate determinations and error bars represent one standard deviation. Representative data is shown from n=2 experiments. B) Scatchard plot showing ^125^I-hIL-3(13-125; W13Y) binding to COS cells transiently expressing IL3Rα only. Data are from a representative binding experiment (n = 4) showing the line of best fit for IL-3 binding. C) Scatchard plot showing ^125^I-hIL-3(13-125; W13Y) binding to COS cells transiently co-expressing IL-3Rα and βc. Data is from a representative binding experiment (n = 6) showing the line of best fit for IL-3 binding. Binding data for panels B and C were analyzed using EBDA-LIGAND software (KELL).

Having established a high-yielding strategy to express and purify soluble hIL-3(13-125; W13Y) from *E. coli*, we proceeded to compare its potency as a growth stimulus in TF-1 cell proliferation assays relative to recombinant hIL-3 sourced from two different commercial vendors. TF-1 is a factor-dependent cell line that endogenously expresses the hIL-3 receptor, and is a cell line commonly used to assay hIL-3 bioactivity. As shown in Figure 3A, in the absence of hIL-3, no TF-1 cell proliferation was observed, illustrating the cell proliferation relies on hIL-3 stimulation. Preparations of hIL-3(13-125; W13Y) purified by SEC (open squares) and by SEC plus RP-HPLC (open circles) were equipotent stimuli in TF-1 proliferation assays (Figure 3A). These preparations of hIL-3(13-125; W13Y) exhibited comparable, if not slightly enhanced, potency compared to hIL-3 sourced from vendor A (black circles). Surprisingly, however, a second commercially-sourced sample (black squares; vendor B) was an order of magnitude less potent than each of our hIL-3(13-125; W13Y) preparations and the hIL-3 supplied by vendor A. Consequently, we concluded that not only are the bioactivities of our SEC product and our RP-HPLC product virtually indistinguishable, but their bioactivities in TF1 proliferation assays were equivalent or better than commercially-supplied hIL-3 from two different vendors.

### Receptor binding assays with radio-iodinated hIL-3(W13Y; 13-125)

We next examined the capacity of hIL-3(13-125; W13Y) to be radio-iodinated for receptor binding studies. We employed a conventional tyrosine-labeling strategy based on that of Fraker and Speck [27], using the protein iodination reagent previously known as Iodogen, to incorporate ^125^I into hIL-3(13-125; W13Y) to high specific activities, typically around 150,000 cpm/ng. We then examined the capacity of ^125^I-labelled hIL-3(W13Y; 13-125) to bind hIL-3 receptors, which we transiently overexpressed in COS cells. While COS cells expressing the hIL-3Rα subunit alone bound ^125^I-hIL‑3(13-125; W13Y) with a K_d_ of 143nM (Figure 3B), as expected, those co-expressing both the hIL-3Rα and hβc subunits bound ^125^I-hIL‑3(13-125; W13Y) with a high-affinity K_d_ of 456pM (Figure 3C).

## Discussion

Since the human IL-3 gene was cloned in 1986 [28], high-yielding, cost-effective approaches have been sought for the expression and purification of hIL-3 for biochemical, structural and biological studies, including as a growth stimulus for cell culture applications. While several different preparation methods from a variety of expression hosts have been described [29–32], *E. coli* remains the most rapid, accessible and inexpensive source of recombinant cytokines. Commonly, however, the preparation of recombinant cytokines from *E. coli* necessitates time-consuming and cumbersome oxidative refolding steps (*e.g.* [33]). In the present work, we demonstrate the efficacy of producing hIL-3 as a soluble fusion protein bearing a NusA-His_6_ solubility/purification tag, which allows for convenient purification prior to cleavage with TEV protease. Consistent with an emerging role for the NusA tag as an alternative to GST fusions to enhance the solubility of poorly-soluble proteins [34,35], we have previously found that the NusA tag was effective in allowing the preparation of mouse IL-3 as a soluble protein from *E. coli* [18] (Figure 1) with yields and solubility in aqueous solvents sufficient for NMR spectroscopy studies [36–38]. In addition to maintaining hIL-3 in soluble form, the present method allows the preparation of milligram quantities of hIL-3 per litre of shaking *E. coli* culture, is rapid, simple and completely avoids refolding. To minimize losses during chromatographic steps, rather than dialyzing the TEV protease-cleaved hIL-3 and performing further Ni^2+^-chromatography, the cleavage reaction was concentrated and applied directly to a preparative SEC column. The large molecular weight difference between the NusA-His_6_ tag (55 kDa) and hIL-3 (13.4 kDa) enabled resolution of the two species by SEC (Figure 2B). Whilst the SEC product was highly purified (Figure 2C) with no detectable NusA or TEV protease present, an additional polishing step, reversed-phase HPLC, can be employed to purify the hIL-3(13-125; W13Y) to homogeneity (Figure 2D). Both the SEC and the RP-HPLC products exhibited comparable bioactivities in stimulating proliferation of the hIL-3-dependent cell lines, TF-1 (Figure 3A). We consider the RP-HPLC step non-essential for structural and biochemical studies of hIL-3, but note it is likely to be advantageous for biological studies since RP-HPLC is known to largely eliminate lipopolysaccharide (LPS) [39], an agonist of the pro-inflammatory TLR4 receptor, which is a common contaminant of proteins prepared from Gram-negative bacterial hosts.

In designing our hIL-3 expression construct, we introduced a tyrosine in place of Trp13 (highlighted in Figure 1) to serve as a substrate for ^125^I-labelling. Wild-type hIL-3 is a poor substrate for commonly employed tyrosine-radioiodination methods, such as Iodogen or iodine monochloride labeling, owing to the sole tyrosine being buried in the helical bundle core ( [26]; PDB, 1JLI), leading to low specific activities when native hIL-3 is tyrosine-radioiodinated [23]. We reasoned that introduction of a tyrosine residue peripheral to the four-helix bundle would overcome a longstanding technical hurdle and allow efficient radiolabelling of the hIL-3 analog. As anticipated, introduction of a non-native Tyr residue N-terminal to the hIL-3 four helix bundle does not impact the bioactivity (Figure 3A). We found that hIL-3(13-125; W13Y) is an excellent substrate for ^125^I-labeling using the Iodogen method and we successfully used the radiolabelled product in receptor binding studies, affording K_d_ values comparable to those reported in prior hIL-3 receptor binding studies [40].

In summary, herein we describe a simple and inexpensive procedure for preparation of a hIL-3 analog as a soluble protein, which exhibits wild-type bioactivity and improved radio-labeling capacity, from *E. coli*. Not only does this procedure typically yield >3mg per litre of shaking culture, but the whole procedure can be completed within 3 days without the necessity of oxidative refolding.

## Supporting Information

Table S1 **Tryptic peptide mass spectra identifying hIL-3(13-125; W13Y).**
High quality spectra identified 89% of the full length construct sequence using Mascot 2.4 upon searching the UniProt database (with the vector-derived overhang, GAMGS, included). Complete details of the experiment are shown in Materials and Methods.
(XLS)
